# Current Challenges to the United States' AIDS Drug Assistance Program and Possible Implications of the Affordable Care Act

**DOI:** 10.1155/2013/350169

**Published:** 2013-03-18

**Authors:** Kathleen A. McManus, Carolyn L. Engelhard, Rebecca Dillingham

**Affiliations:** ^1^Department of Medicine, University of Virginia, Charlottesville, VA 22903, USA; ^2^Department of Public Health Sciences, University of Virginia, Charlottesville, VA 22908-0717, USA; ^3^Division of Infectious Diseases and International Health, Department of Medicine, University of Virginia, P.O. Box 801379, Charlottesville, VA 22908, USA

## Abstract

AIDS Drug Assistance Programs, enacted through the Ryan White Comprehensive AIDS Resources Emergency Act of 1990, are the “payer of last resort” for prescription medications for lower income, uninsured, or underinsured people living with HIV/AIDS. ADAPs face declining funding from the federal government. State funding of ADAP is discretionary, but some states increased their contributions to meet the gap in funding. The demand for ADAP support is increasing as people living with HIV are living longer; the antiretroviral therapy (ART) guidelines have been changed to recommend initiation of treatment for all; the United States is increasing HIV testing goals; and the recession continues. In the setting of increased demand and limited funding, ADAPs are employing cost containment measures. Since 2010, emergency federal funds have bailed out ADAP, but these are not sustainable. In the coming years, providers and policy makers associated with HIV care will need to navigate the implementation of the Affordable Care Act (ACA). Lessons learned from the challenges associated with providing sustainable access to ART for vulnerable populations through ADAP should inform upcoming decisions about how to ensure delivery of ART during and after the implementation of the ACA.

## 1. Introduction

AIDS Drug Assistance Programs (ADAPs) were enacted through the Ryan White Comprehensive AIDS Resources Emergency (CARE) Act of 1990. These programs, an integral component of HIV care in the United States, are the “payer of last resort” for prescription medications for lower income, uninsured, or underinsured people living with HIV (PLWH). The provision of these medications to all provides an important incentive for HIV testing, as testing agencies can assure those tested that treatment will be available regardless of ability to pay. In addition, an uninterrupted supply of subsidized HIV medications provides the best opportunity for enrolled individuals to maintain an undetectable viral load, reducing their risk of transmitting the virus. In 2006, ADAP accounted for 41% of the CARE Act's $1.93 billion budget [1]. According to the National Association of State and Territorial AIDS Directors, ADAP provides ART to 1 in 4 PLWH, indicating that the “safety net” is holding a substantial population. Unfortunately, ADAPs have not been able to meet the demand for their support, and they recently faced the worst funding crisis since their inception [2]. ADAP funds vary based on annual federal allocations and discretionary state support, making planning difficult. With the Affordable Care Act (ACA) coming into effect, there are many questions about how its provisions will affect ADAP's role in delivering essential medications to PLWH.

## 2. Federal Government

The federal share of the national ADAP budget has declined from a high of 68% in 2000 to 43% in 2011 [3]. Between 2004 and 2009, the CARE Act underwent its third reauthorization. For ADAP, this introduced a new formula for determining funding to states. This formula incorporated the number of living HIV cases into the calculation rather than only counting cases with AIDS. The reauthorization, or “modernization,” also set forth a new minimum formulary requirement [4]. Unfortunately, the funds allocated to ADAP through the “modernized” CARE Act have not met the growing need for subsidized HIV medications. 

In 2004, President Bush allocated $20 million in one-time funding to address ADAP waiting lists in 10 states as part of the President's ADAP Initiative [4]. These funds were specifically earmarked for the 1,738 clients who were on the wait lists at the time of the announcement [5]. However, by April 2005, 21 ADAPs reported having one or more cost containment measures in place, including wait lists in 11 states representing a total of 627 individuals [5]. In August 2010, the Obama Administration acknowledged the continuing crisis by allocating $25 million in funding to decrease ADAP waiting lists and ease cost containment measures. This emergency funding was distributed to 30 states. Despite this allocation, from August 2010 to August 2011, the national ADAP wait list swelled from 2,937 to 9,217 [6, 7]. In September 2011, an additional $40 million in emergency federal funds was allocated to ADAPs with wait lists. This made a dent in the wait list numbers with a 28% decrease over 3 months and then a further 18% decrease over the next 6 months [8, 9]. However, while states were retrieving individuals from their wait lists and placing them back on their ADAP rolls, they also continued to add new individuals to their programs, in part due to expanded HIV testing. Therefore, on December 1, 2011, World AIDS Day, President Obama announced an additional $35 million in ADAP funding with the goal of enrolling 3,000 people from the ADAP wait lists [10]. In July 2012, the funds were released, and all states were funded with the amount requested [11]. As of November 2012, only 3 ADAPs have individuals on their wait lists. While this is a huge step in the right direction, 2 ADAPs report having capped enrollment, and one anticipates opening a wait list before the end of the fiscal year [12]. This demonstrates that ADAP enrollments and wait lists are not static. The financial health of ADAP, its ability to maintain its commitment to enrolled patients, and its ability to keep up with new demand remain dependent on large emergency disbursal of federal funds. This level of financial uncertainty threatens the viability and the effectiveness of ADAP for the individuals it serves and the population as a whole.

## 3. States

General revenue support from state budgets is, for the most part, dependent on individual state decisions and budgets. State governors are struggling to balance budgets, and states are generally not required to allocate funds to ADAP. Nonetheless, state funding accounted for $306 million or 16% of the ADAP budget in FY 2011 [3]. From 2009 to 2010, states had increased their contribution by 61%, and they provided an additional increase of 9% from 2010 to 2011 [3, 4]. Despite individual states' efforts to fill the gap left by decreasing federal funds, state budgets may face more difficult times as federal one-time stimulus and other special funds that had been used to sustain the ADAP program run out. In addition, budget gimmicks, such as deferring major expenses, cannot be sustained. 

## 4. Industry Partners

Since 2003, the ADAP Crisis Task Force (ACTF) has negotiated with manufacturers to obtain reduced medication prices. With agreements with 12 pharmaceutical partners, the ACTF secured a savings of $259 million in 2009 for ADAP. The cumulative savings since 2003 has been more than $1.2 billion [13]. After the passage of the ACA, ACTF met with pharmaceutical partners in May 2010 to assess if any further discounts could be granted due to the worsening financial status of ADAP. ACTF and the pharmaceutical industry came to an agreement that has been described as a “bridge to 2014.” It includes deeper discounts, increased rebates, and price freezes for ADAP. It also includes plans to enhance Patient Assistance Programs (PAPs). PAPs are pharmaceutical-sponsored and provide free or discounted medication to lower income, uninsured, and underinsured people who meet the guidelines. It was estimated in July 2010 that the new agreements will reduce ADAP's annual antiretroviral costs by $160 million, which is a savings of about 18% [13]. Since 2011, the ACTF reached agreements to further reduce costs with Boehringer Ingelheim, Gilead Sciences, Janssen Therapeutics, Bristol-Myers Squibb, Merck, and ViiV Healthcare [11].

## 5. Increasing Demand for ADAP Support

ADAPs are seeing increased demand for their support. In less than a decade, from 2003 to 2011, the number of ADAP clients ballooned from 128,465 to 226,419 [3]. Many factors have contributed to the increased client load. PLWH are surviving longer, and once they are started on ART, it is continued for life. In addition, previously, ART was started when a patient's CD4 count was below 350, but the Department of Health and Human Services (HHS) recently recommended starting ART regardless of CD4 count [14]. Many states have not been able to supply ADAP support to all patients who need ART under this revised standard of care. 

There has also been increased emphasis on testing people for HIV as the CDC estimates that of the 1,178,350 Americans living with HIV, approximately 240,000 (or 21%) are unaware of their HIV-positive status [15]. The CDC supports opt-out testing for HIV for all people aged 13–64 years [16]. In 2007, the CDC started an Expanded Testing Initiative, which has focused on increasing HIV testing in populations disproportionately affected by the disease. The Ryan White HIV/AIDS Treatment Extension Act of 2009 included a “National HIV/AIDS Testing Goal” of 5 million tests per year [17]. With increased diagnoses and the goal of treating all people with HIV, ADAP's sustainability is challenged [18]. When access to subsidized treatment is threatened, ethical dilemmas arise. Providers might begin to consider whether or not to test patients if they will not have access to treatment [19, 20]. In addition, the presence of wait lists, or an inability to access antiretroviral medications, may reduce a person's motivation and ability to engage in HIV care [21].

In addition to increasing numbers due to changes in guidelines and testing, economic pressures are also swelling the ADAP rolls. As indicated earlier, at least 23% of PLWH already receive their medications through ADAP [22]. As the recession continues, more PLWH face economic hardship and fall within the income range eligible for ADAP but are ineligible for other public assistance for their healthcare. Just as the number of individuals needing help with therapy is increasing, so is the cost of the therapy. A 2006 study estimated that people living with HIV who initiate ART according to guidelines will spend over $450,000 on medications alone in their lifetime [23]. Although calculated just five years ago, this cost estimation is outdated as more patients are treated earlier based on new guidelines and as more patients utilize expensive salvage regimens.

## 6. ADAP Cost Containment

With increasing enrollment and uncertain funding, cost containment in ADAP programs has been necessary. Examples of cost containment measures include wait lists, lowered financial eligibility, changed medical criteria for enrollment, reduced formulary or tiered formulary, annual/monthly expenditure cap, clients disenrolled for not accessing ADAP for 90 days, capped enrollment, client cost sharing, and patients transitioned to PAPS [5].

Wait lists have been particularly problematic, especially in reference to equitable referral to and from the wait lists. With Massachusetts data from 2003, Linas et al. demonstrated that by using CD4 cell count-based enrollment criteria rather than a first-come, first-served, ADAPs served more diverse populations and patients with significantly more advanced HIV disease [24]. Another study by Linas et al. showed that when faced with limited resources, ADAP could improve outcomes by prioritizing patients with lower CD4 counts [25]. However, this strategy does not align with current HHS guidelines [14].

In September 2011, with the disbursal of the emergency ADAP funds, states immediately began to try to reduce their restrictions on accessing medications. For example, Virginia received $3 million in ADAP funds earmarked specifically to reduce its wait list. With these funds, Virginia was able to enroll persons from the wait list into ADAP for the first time since the institution of a wait list. The state used a medical criteria model to enroll, prioritizing those with CD4 counts under 200 first and then moving to those with higher CD4 counts [26]. In July 2012, the state was able to enroll all those on the wait list regardless of CD4 count. 

In addition to implementing wait lists, some states have limited the medications available through their ADAPs. Each state's ADAP determines the composition of its formulary. Since there is no federal mandate regarding ADAP coverage for non-HIV medications, there is a wide variation in the medications for comorbidities that are provided by ADAP [27]. In November 2010, for example, Virginia's ADAP eliminated all medications from the ADAP formulary that were not antiretrovirals, vaccines, or treatments for opportunistic infections. This included removing medications for common comorbidities like hypertension, hyperlipidemia, diabetes, and mental health conditions. With the emergency federal funds that were disbursed in summer 2012, Virginia was able to restore its formulary.

## 7. ADAP Cost Containment and Gaps in Knowledge

The effects of ADAP cost containment measures on the United States HJV population have not been evaluated. Interruptions in HIV care need to be quantified at the clinic, state, and national level, and if present, their effects on engagement in care and on HIV control, including viral load and CD4 count, should be studied. Losing access to medications may discourage PLWH from pursuing care at all, which places the individuals' lives at risks as well as those of their partners and other contacts. Moreover, the consequences of constrained access to medications for comorbidities also require assessment since people with HlV are living longer, and poor control of comorbid conditions poses increasing health risks. Recently, it has been estimated that 10% of deaths in PLWH are due to cardiovascular disease [28]. Treating diabetes, hyperlipidemia, and hypertension are important parts of decreasing cardiovascular risk factors which may be exacerbated by HIV infection itself and/or by ART [29]. While the recent reduction in ADAP wait lists is encouraging, the combination of constrained and uncertain federal and state resources coupled with a growing demand for care and costly medications suggests that rationing of ART and other key medications will continue, resulting in a failure to meet current standards of optimal HIV care in the US. 

## 8. ADAP and ACA

The ACA is poised to change healthcare for many Americans including people living with HIV. With the ACA, it is hoped that patients may have more options for adequate prescription coverage under the insurance coverage expansion, leaving ADAP as the true “payer of last resort” for HIV medications. Consumer protections under the ACA began in 2010, when the ACA was initially rolled out. In late September, 2010, health plans were prohibited from placing lifetime limits on coverage. These consumer protections will become stronger in January 2014, with bans on denying coverage to adults with preexisting conditions and bans on annual dollar limits of coverage. This is valuable to people living with HIV who can have high health costs, most of which are due to prescription medication expenses [22].

In March 2010, with the passage of the ACA, ADAP, as a 340B program, became eligible for reduced price medications. The federal 340B Drug Discount Program, authorized under the Veterans Health Care Act of 1992, allows federal agencies to purchase most drugs at or below the 340B ceiling price, which results in saving between 20% and 50% [30]. With this, ADAP was able to purchase medications at even greater price reductions. 

Prescription medication coverage has also expanded under the ACA for those PLWH eligible for Medicare. In the past, Medicare did not count ADAP funds towards a patient's true-out-of-pocket (TrOOP) costs. Achieving a certain level of TrOOP costs allows a beneficiary to receive Medicare catastrophic drug coverage. Effective January 2011, the ACA started allowing ADAP spending on HIV drugs to count towards TrOOP costs as well. This stretches ADAP resources farther. Previously, once a Medicare beneficiary entered the Medicare prescription coverage gap or “donut hole,” ADAP would help to defer costs, and the beneficiary required ADAP's help until a new insurance year started. Now, ADAP can help a Medicare beneficiary through the “donut hole” until Medicare catastrophic coverage begins, leaving ADAP funds free for others [22].

As part of the more permanent fix, the ACA has begun to phase out the “donut hole” in 2011 with plans for its elimination by 2020. In the meantime, the ACA gave a $250 rebate to Medicare beneficiaries who reached the drug coverage gap in 2010; and starting in 2011, it provided a 50% discount on brand-name drugs and a 7% discount on generic drugs. These discounts are scheduled to increase each year until the gap is eliminated in 2020 [22].

While the changes that have already started to take effect may help PLWH, many look towards 2014 and the Medicaid expansion to help more with healthcare and prescription coverage for vulnerable PLWH. Currently, childless people with HIV must be classified as disabled in many states in order to qualify for Medicaid. Other Medicaid eligible categories include poor children, parents with dependent children, and pregnant women. However, states vary with regard to eligibility because states can choose to exceed federal income thresholds. Medicaid is currently the largest provider of HIV care. Forty seven percent of PLWH, who are engaged in care, are already covered by Medicaid [31]. The federal government has offered to fund a portion of the Medicaid expansion, which will increase income cutoffs to 138% of the federal poverty level (FPL) and remove the categorical eligibility requirement. Therefore, any PLWH (or anyone else) who fulfills the income criterion will be eligible in those states that choose to accept the federal funds for the expansion. However, the expansion is not mandatory, and, of this writing, only 17 states have indicated a desire to expand their Medicaid program, despite the federal government's offer to pay 100% of the new costs over the first three years, and over 90% of the costs into perpetuity. Approximately half of all people who receive ADAP support have incomes under 138% FPL and could potentially become Medicaid beneficiaries [22]. The other half will need to enter the ACA-enabled health insurance exchanges to receive prescription coverage, and if their income is 100% to 400% FPL, they will qualify for subsidies in the form of tax breaks to help in the purchase of insurance [22].

## 9. Looking Forward with ADAP

It is in the government's best interest to have a stable, sustainable resource to provide ART to PLWH. Providing ART to patients is an important public health issue because it lengthens a patient's survival, and it decreases a patient's chance of transmission to a sexual partner [32–35]. Mathematical models suggest that the successful use of ART can reduce the spread of HIV at the population level [36, 37]. Establishing adequate access to ART for all people living with HIV is now considered a top priority in the CDC's HIV prevention plan, and as such, the program should be prioritized. While the ACA will increase the number of individuals with access to healthcare, ADAPs will likely still be necessary as a safety net for socioeconomically vulnerable PLWH. Given the high importance of this program to individual and public health, it should be prioritized and carefully monitored along the lines suggested after.

With both federal and state governments looking for areas to cut in order to meet balanced budgets, ADAPs remain vulnerable [13]. It is in the best interest of PLWH and the public, for PLWH to be engaged in care with maximum viral suppression. This maximizes a PLWH's health and minimizes the risk of transmitting the virus. Only with guaranteed coverage through universal healthcare or through HIV becoming an entitled group under Medicare, like patients with end stage renal disease, will PLWH become a less vulnerable population. 

In the meantime, continued discussions with pharmaceutical industry partners will also be essential to help defray costs to ADAP. Continued discussions with pharmaceutical industry partners will be essential to help defray costs to ADAP. The pharmaceutical industry gains from its partnership with ADAP because of the ADAPs' large market share and the large profit margin on medications. In 2010, many of the ART manufacturers individually reported over $10 billion in profits [38]. In the face of the recent ADAP funding crisis, the pharmaceutical industry could be asked to further discount ART. 

The cost containment practices of state ADAPs also need to be evaluated. The sporadic restrictions burden patients, physicians, and Ryan White clinic staff. For example, in Virginia, in November 2010, 247 ADAP clients were disenrolled due to funding shortages [39]. Ryan White Clinic staff scrambled to find alternative sources of medications for patients. The Virginia Department of Health said that they would continue to supply the disenrolled ADAP patients with ART until they had established other means of obtaining their medications. However, there have been reports of patients being dropped from ADAP before they were able to secure medications from alternative sources [40]. While not HIV-specific, a study of patients with chronic illness in Oregon who were on public insurance demonstrated that changes in health care insurance plans created interruptions in coverage that culminated in reduced quality of life and worsened disease outcomes [41]. Given the potential physical harms, both acute and chronic, of ART interruptions as well as the stress they cause patients, a better understanding of their frequency and effects are needed. Better procedures to avoid coverage gaps that may result in interruptions are essential [42].

Continued monitoring of the state and federal funding of ADAP, particularly by organizations like NASTAD and the Kaiser Family Foundation (KFF), is critical. Through the work of these organizations, ADAP's enormous role in American HIV care, despite having been conceptualized as a “safety net,” is clear, as is the crisis of funding. Ethically, it is wrong to actively increase HIV testing while there is limited access to the standard of care for low income, underinsured, and uninsured patients. While President Obama's allocation of $75 million in emergency funding for ADAPs is impressive and has reduced the ADAP wait lists for the moment, we need a more sustainable method for providing ART for lower income people living with HIV. It has been estimated that the amount needed to address unmet ADAP needs is $360 million [3]. Financially, using a “safety net” program with flat funding for up to a third of HIV patient care is not feasible. We have reached a ceiling in federal funding and states do not have the resources to maintain ADAP at its current level and expand it to fulfill future needs. In terms of public health, inadequate access to ART means that there is likely to be increased transmission of HIV. From a medical standpoint, there are many people living with HIV in the United States who are not receiving the standard of care, which currently means starting ART at diagnosis of HIV.

As mentioned earlier, with the continued implementation of the ACA, it is hoped that patients may have more options for adequate prescription coverage, leaving ADAP as the true “payer of last resort” However, there are many unknowns in terms of how the ACA will change the current insurance and medication benefits of PLWH, and it is possible that some services for PLWH could be marginalized even as other services expand. 

For example, if Medicaid is expanded nationally to help this population, the expanded Medicaid formularies need to be examined. There are concerns that the formularies supported by expanded Medicaid and by the new health benefit exchange insurance policies will not be sufficient for PLWH. Treating HIV often requires complicated, tailored combinations to achieve viral suppression [22]. HHS has made it clear that it will not use the Medicare Part D formulary, which is unfortunate. For HIV care, the Medicare formulary would be a good model for the new formulary. ART is one of its protected six categories of medications, and it is unconditionally covered. If a state-based Medicaid or health benefit exchange formulary is too restricted and does not cover multiple medications within different ART classes, patients' ART regimens will not be consistent with the clinical standard.

Twenty-six states challenged the constitutionality of the Medicaid expansion while thirteen filed briefs in favor of the Medicaid expansion (2 were on both sides) [43]. As of December 2012, only four of the 17 states that have opted in to the ACA-expanded Medicaid are on the list of the 20 states that have had ADAP waiting lists at some time since January 2010 [22]. Traditionally, the states that do not cover childless adults (and which have not begun an expansion of Medicaid) are the states that already have strained Ryan White (and therefore ADAP) funds. Ryan White funds will be reappropriated in 2013. If the programs are not fully funded, it is possible that people living with HIV in the 20-plus states without the Medicaid expansion will face difficulties because there will be no new safety net for poor PLWH. Instead, new patients or newly poor or uninsured patients will continue to rely exclusively on ADAP. Even if the Medicaid expansion becomes nationwide over time (as was the experience with the original Medicaid program implemented in the mid-1960s), there is concern that there will be a new gap in coverage for people who cannot get access to Medicaid and who have incomes too high to qualify for health exchange subsidies [22]. Also, it is unclear if tax credits will be enough incentive for this population to buy insurance, despite the individual requirement under the ACA to carry insurance, since the out-of-pocket costs may be high. It is also unclear if the tax penalty under the ACA for failure to purchase health coverage ($95 or 1% of income in the first year) will be enough of a punishment to incentivize purchasing of insurance, especially if the penalty is less than the price of insurance.

Besides thinking about how many states will expand Medicaid, we also need to think about how this vulnerable population will be enrolled. It will require staff and resources with targeted outreach efforts to enroll this population. In addition, enrolling these many new beneficiaries will be time intensive for both government officials as well as recipients. Eligibility will likely be determined more than once a year, and beneficiaries will need to locate necessary financial documentation. Unfortunately, studies have shown that public insurance policy changes often result in disenrollment of chronically ill patients [41].

Moreover, it has been shown that, in the population of PLWH, patients with public insurance were twice as likely to miss clinic appointments within the first year of care than patients with private insurance or no insurance [44]. In univariate analysis, public insurance was also associated with two times the mortality rate at one year when compared with the private or no insurance populations [44]. It is likely that the population of HIV patients with no insurance were receiving care through Ryan White Funds. This suggests that even if more patients with HIV are enrolled in Medicaid, it may not improve outcomes. The reasons for their discrepancy are not clear and require further study, but one hypothesis is that the comprehensive care provided under the Ryan White ADAP program may provide better treatment options and coordination, which may lead to better outcomes. Given the complex individual care needed and public health risks at stake, Ryan White and ADAP-like services, rather than an expanded Medicaid or private insurance program, may be necessary to achieve the best outcomes for people living with HIV.

There are many opportunities for positive changes in the US health system over the next few years, but the ACA will likely not resolve all the complex issues faced by PLWH. We will need to watch for unintended consequences as PLWH shift into public and private health programs under the ACA. Of particular concern will be whether the transition into Medicaid and private insurance effectively shortchanges coordination of care even as it expands coverage. To this end, it is vitally important to ensure that Ryan White and ADAP have adequate funding to support their safety net programs as we monitor and measure the changes ahead.
